# Long Non-Coding RNAs and Their Potential Role as Biomarkers in Inflammatory Bowel Disease

**DOI:** 10.3390/ijms25168808

**Published:** 2024-08-13

**Authors:** Lorena Ortega Moreno, María Chaparro, Javier P. Gisbert

**Affiliations:** 1 Área Farmacología, Bromatología y Nutrición, Departamento Ciencias Básicas de la Salud, University Rey Juan Carlos (URJC), 28922 Alcorcón, Spain; 2 High Performance Research Group in Physiopathology and Pharmacology of the Digestive System (NeuGut), University Rey Juan Carlos (URJC), 28922 Alcorcón, Spain; 3 Gastroenterology Department, Hospital Universitario de La Princesa, Instituto de Investigación Sanitaria Princesa (IIS-Princesa), Universidad Autónoma de Madrid (UAM), and Centro de Investigación Biomédica en Red de Enfermedades Hepáticas y Digestivas (CIBERehd), 28006 Madrid, Spain; mariachs2005@gmail.com (M.C.); javier.p.gisbert@gmail.com (J.P.G.)

**Keywords:** long non-coding RNAs, inflammatory bowel disease, Crohn’s disease, ulcerative colitis, biomarkers

## Abstract

Inflammatory bowel disease is a chronic inflammatory disease that encompasses entities such as Crohn’s disease and ulcerative colitis. Its incidence has risen in newly industrialised countries over time, turning it into a global disease. Lately, studies on inflammatory bowel disease have focused on finding non-invasive and specific biomarkers. Long non-coding RNAs may play a role in the pathophysiology of inflammatory bowel disease and therefore they may be considered as potential biomarkers for this disease. In the present article, we review information in the literature on the relationship between long non-coding RNAs and inflammatory bowel disease. We especially focus on understanding the potential function of these RNAs as non-invasive biomarkers, providing information that may be helpful for future studies in the field.

## 1. Introduction

Inflammatory bowel disease (IBD) is a chronic inflammatory disease that encompasses entities such as Crohn´s disease (CD) and ulcerative colitis (UC). Currently, IBD is incurable and is mainly diagnosed in early adulthood [1]. Its incidence has increased over the years in newly industrialised countries, turning IBD into a global disease [2]. Moreover, its prevalence in developed countries has remained high over time; taken all together, health-care systems may not be prepared to cope with the increasing burden of IBD [3]. In this scenario, treatments have evolved with the introduction of drugs that have contributed to reductions in the need for surgery and improvements in the quality of life of patients; unfortunately, the costs of these biological treatments are very high [4]. Accordingly, new strategies to reduce the burden of IBD are necessary. Recently, studies on IBD have focused their efforts on finding non-invasive and specific biomarkers. Long non-coding RNAs (lncRNAs) may play a role in the pathophysiology of IBD and therefore these biomolecules can be considered to be good candidates for biomarkers for these diseases [4].

Our aim was to review the recent literature on the role of lncRNA in IBD to provide useful information for future studies on lncRNAs as therapeutic targets or potential biomarkers for IBD.

An electronic search was performed in PubMed using the following keywords: (lncRNA OR non-coding RNA) AND (inflammatory bowel disease OR Crohn’s disease OR ulcerative colitis). This search covered publications from January 2009 to June 2024. We have emphasised the review of publications from the last five years.

## 2. Non-Coding RNAs: A Brief Description

**Figure 1:**
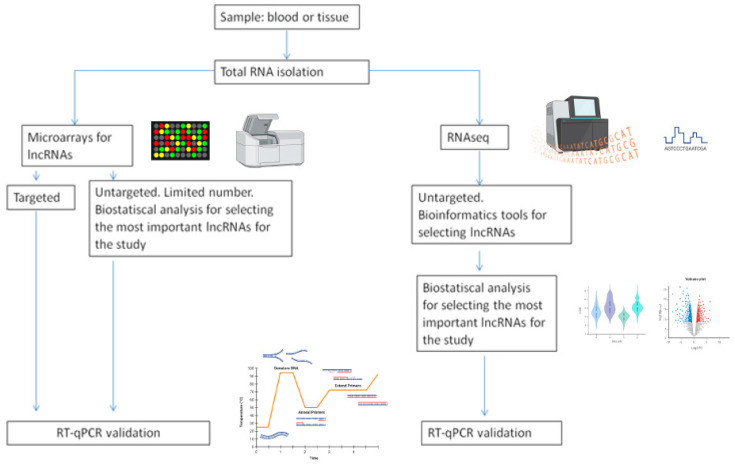
Workflow of lncRNAs studies (some parts of this figure were made with the help of Biorender).

Until a few years ago, non-coding RNAs (ncRNAs) were considered transcriptional “noise”. However, the recent use of advanced technological tools has led to the knowledge that these ncRNAs constitute most of the transcripts of the human genome [5]. There are different methods for lncRNAs isolation (Figure 1).

Only 4% of the human genome codes for proteins, while approximately 85% of the genome is transcribed into RNA. Conceivably, improving our understanding of the genomic regions coding for ncRNAs may be crucial to efforts to understand more deeply the genetic causes of diseases [5].

Recently, advanced transcriptomics studies have allowed the investigation of non-coding polynucleotides such as ncRNAs and their relationship with disease, particularly with chronic disease. The ncRNAs have emerged as potential disease biomarkers, as they are easy to obtain from several clinical specimens, and highly tissue- and cell type-specific [6]. 

Most ncRNAs are microRNAs, lncRNAs and circular RNAs (circRNAs) [7]. Briefly, microRNAs bind to a sequence located in the 3’UTR region of the mRNA, and their length is about 200 nucleotides. These micro RNAs can interact with lncRNAs; in fact, lncRNAs could sequester microRNAs and regulate their abilities [7].

CircRNAs are originated in the process of back-splicing and contain exons, introns and non-coding intergenic regions [7] This kind of ncRNAs can bind to proteins [8] or act as a microRNA sponge [9], similarly to lncRNAs.

The major type of ncRNA is lncRNA. LncRNAs are non-coding RNA molecules longer than 200 nucleotides [10]. They are diverse and numerous, outnumbering protein-coding RNAs [11].

Several lncRNAs show mRNA-like characteristics, such as a polyA tail and splicing, that allow lncRNAs to exit the cell nucleus and enter mRNA pathways. Despite these similarities, lncRNAs are less well conserved than mRNAs [12] but are more tissue-specific and work at much lower concentrations [13]. More than 80% of lncRNAs effect potentially important activities such as protein binding [5]. Although they bind proteins, the functional relevance of protein–lncRNA interactions remains to be elucidated.

The limited sequence conservation between lncRNAs from different organisms suggests that they may not be functional across organisms. Nonetheless, lncRNAs exert actions requiring conservation of small portions of the sequence [14]. Standard detection methods are not adequate to analyse these portions of the sequence; hence, this analysis requires adaptation of existing bioinformatic tools and development of new methods focused on lncRNA promoter regions [15]. Although sequence conservation of lncRNAs is low, their promoter regions are as strongly conserved as promoter regions of protein-coding genes [16]. Therefore, lncRNA conservation is selective and restricted to transcriptional regulation.

There is limited knowledge of the origin of lncRNAs, although major lncRNAs are lineage-specific. A plausible mechanism for lncRNA evolution is the loss of function of coding genes. Another possibility is the association of non-coding regions of the genome with a promoter, forming a functional lncRNA [16].

LncRNAs’ categorisation depends on their structure and location within the cell. LncRNAs may be located both in the nucleus and in the cytoplasm, and they can move from one site to the other in response to signals [17]. Close to the chromatin, they affect gene expression activating or repressing transcription [18]. According to their location within the genome, lncRNAs are considered intergenic lncRNAs (lincRNAs) when they are transcribed from intergenic regions [7] (these do not overlap with other genes), and sense or anti-sense lncRNAs when they overlap with other genes in a sense or anti-sense orientation, respectively. Intronic lncRNAs are those located within introns of other genes. Bi-directional lncRNAs are in the same region as a different gene but in the opposite direction. The most common types are intergenic and anti-sense lncRNAs [19].

Nuclear lncRNAs are able to bind to DNA or nuclear proteins, and some of them may self-interact or compete with DNA [20,21]. Furthermore, weak interactions between lncRNAs and splicing factors contribute to nuclear retention of mRNA by regulating RNA splicing [22].

RNAseq methods have revealed that many lncRNAs are polyadenylated and interact with ribosomes, where they can be translated [23]. Another characteristic of some lncRNAs is their capability to harbour miRNA binding sites [24] that may act as RNA “sponges” capable of regulating mRNA levels. 

In addition, some lncRNAs are contained in extracellular vesicles, and some studies have hypothesised that lncRNAs with low expression may be enriched in extracellular vesicles [25], modulating protein functions or cell viability [26]. 

LncRNAs exert several functions, including shaping chromosome conformation, coordination of cell state, differentiation and development. LncRNAs are present at every stage of gene regulation and may even act as miRNA decoys [27]. They inhibit protein translation, working as post-transcriptional inhibitors [28] or controlling mRNA stability [29]. In addition, functions as scaffolds and guides, and the recently described function of regulation of enzyme function, have also been attributed to lncRNAs [30,31]. Changes related to lncRNA genes, such as overexpression, mutation or deficiency have been implicated in many diseases [12]. 

Furthermore, lncRNAs participate in differentiation, metabolism and physiological functions in tissues and organs. They play physiological roles in nervous, cardiac, pancreatic, intestinal, epidermal, germ line, bone, adipose, hepatic, lung and muscular tissues [32]. It is remarkable that some lncRNAs contain open reading frames encoding functional peptides (e.g., the muscle-specific lncRNA-derived 34-aminoacid micropeptide called DWORF, which enhances muscle contraction) [33].

As we have described above, the functions of many biological lncRNA targets make these molecules good potential biomarkers of disease.

In this review, we focus on the relationship between lncRNAs and IBD and we summarise the recent literature on studies in murine models of disease and in patients, both targeted (studies restricted only to some well-known lncRNAs) and untargeted studies.

## 3. LncRNAs and IBD

We have already mentioned the importance of finding new non-invasive biomarkers for the diagnosis and prognosis of IBD. Several biomolecules or cell types have been proposed as potential biomarkers in IBD, such as adipokines, immune blood cells and peptide profiles obtained by proteomics techniques [34,35,36]. Notably, polynucleotide biomolecules as lncRNAs currently stand out among these potential biomarkers.

### 3.1. Murine Models 

Murine models contribute to a deeper understanding of IBD pathophysiology. The most widely used mouse model for IBD is dextran sulphate sodium (DSS)-induced colitis. RNAseq analysis of colonic tissue from two mouse models of colitis and subsequent RNA validation by RT-PCR identified three lncRNAs with increased expression in mice with colitis [37]. Comparison of these results with gene expression profiles and results from UC patients showed that one of these lncRNAs was also increased in IBD in humans [37]. This study detected 1829 lncRNAs in the mouse colon by RNAseq and, among them, there were 15 lncRNAs differentially expressed [37]. Changes in the expression of mRNAs and lncRNAs are involved in the regulation of the intestinal epithelial barrier in DSS-treated mice [37]. In this regard, Yang et al. showed that the lncRNA CRNDE promoted epithelial cell apoptosis in mice with colitis and found that reduction of CRNDE levels ameliorated colitis symptoms, suggesting that this lncRNA could be a treatment target for IBD [38]. Taken altogether, these studies suggest that a network of lncRNAS, miRNAs and mRNAs that interact with each other may affect the epithelial intestinal barrier in IBD. 

A study of the lncRNA H19 in intestinal epithelial cells in mice with DSS colitis concluded that H19 is an lncRNA involved in an inflammatory pathway linking IL22 to cell growth regulation which plays a role in intestinal epithelial regeneration under inflammatory conditions [39]. H19 gene transcripts changed in the small intestine in response to sepsis: LPS activated intestinal H10 expression in both sexes. Therefore, under inflammatory conditions H19 is expressed in intestinal epithelial cells in vivo. In vitro studies revealed that IL22 induced H19 expression in intestinal epithelial cells; the authors investigated the pathways of IL22, and found an effect of a protein kinase activator, forskolin, on H19 expression in the cell line HT29, and they obtained similar results for STAT3, which is a common pathway of IL22 in intestinal epithelial cells. Therefore, it might be true that Il22 induces H19 expression via STAT3 and PKA activation [39].

Another interesting lncRNA that protects mice from IBD is CARINH [40], which contributes to gut microbiota homeostasis and controls intestinal inflammation. This lncRNA is highly expressed in immune cells and in mucosal tissues, including the intestine, in both mice and humans [40]. In fact, Carinh and the gene Irf1 protect the host against colitis. The Carinh/Irf1 relationship is sustained by microbial factors, and when colitis appears, Carinh/Irf1 regulates the induction of the anti-inflammatory factor IL-18BP [40].

Higher expression levels of the lncRNA NEAT1 have been detected in intestinal tissue of DSS-treated mice, and in this model NEAT1 is involved in the inflammatory response by contributing to the maintenance of the intestinal epithelial barrier function [41]. Another study in mice investigated the role of NF-kB-specific lncRNAs in the regulation of inflammation networks [42]. This study showed that NEAT1 promoted inflammation in intestinal tissue by inducing the translocation of NF-kB- p65 to the nucleus. NEAT1 expression was upregulated in UC tissues and promoted NF-kB- p65 translocation through upregulation of TNFRSF1B expression mediated by mRNA stabilization [42]. Overall, these results suggest that NEAT1 could be used as a biomarker and a potential treatment target in IBD. Therefore, further studies in humans are warranted to fully elucidate these roles.

In addition, the lncRNA NAIL has a role in the initiation and progression of colitis in mice. NAIL expression is enhanced in inflamed colon tissues and correlates with NF-kB activity, thereby playing a role in NF-kB-p38-dependent colitis. This lncRNA is conserved across mice and humans and is specifically induced by the cytokines TNF-alpha and TLR4 ligand LPS in a p65-dependent fashion [43]. Accordingly, this lncRNA could be a potential biomarker, as well as a therapeutic target, for IBD.

The potential therapeutic effect of melatonin on IBD has been studied at the transcriptional level in the DSS colitis model. An RNA sequencing study in dendritic cells from DSS mice concluded that melatonin treatment delayed maturation of bone-marrow-derived dendritic cells in colitis through certain ncRNAs involved in the PI3K-Akt pathway, such as the lncRNA ENSMUST00000226323 [44]. Since dendritic cells are involved in the immune response, their regulation through this mechanism may explain the effect of melatonin on IBD.

An interesting study demonstrated that the lncRNA PCSK6-AS1 promoted T helper 1 (Th1) differentiation and increased epithelial barrier injury, thereby aggravating colitis in mice. This effect of PCSK6-AS1 was mediated by its interaction with a regulation of the kinase HIPK2 [45]. These results open a new possibility for IBD treatment because targeting HIPK2 through PCSK6-AS1 may reduce Th1 differentiation, and therefore protect the intestinal mucosal tissue [45].

Finally, several studies have analysed the relationship between functional food components and IBD. Among them, a recent study reported the beneficial effect of the polyphenol resveratrol on colitis in mice through induction of MUC2 synthesis via the lncRNA ANRIL [46].

### 3.2. Targeted Studies in Human IBD

At the very beginning, microarrays analysis was the most useful technique employed to study ncRNAs, including lncRNAs, although it only allowed for the identification of a limited number of these molecules. Nowadays, the sensitivity of this technique has improved considerably, allowing researchers to perform targeted studies of ncRNA expression.

Both microarray and RNA sequencing data showed that lncRNAs are influenced by immune responses. The majority of these lncRNAs are trans-acting regulators [47].

Padua et al. [48] performed a microarray-based lncRNA expression profiling with colon resection samples from UC patients and healthy controls, identifying lncRNAs differentially expressed in UC. They also evaluated the relationship between these differentially expressed lncRNAs and known IBD genomic alterations, showing that some of these lncRNAs were associated with clinically validated IBD loci. In this regard, the lncRNA IFNG-AS1 was increased in human colitis tissues and was associated with the single nucleotide polymorphism (SNP) rs7134599 located close to the IFNG gene [48]. Furthermore, the murine ifng-as1 gene is also overexpressed in mouse models of colitis [48,49].

Interestingly, an antisense lncRNA (CD39-AS) regulates CD39 mRNA levels in CD, and silencing this lncRNA restores CD39 levels in vitro. These observations are potentially interesting for future CD treatments [50].

LncRNAs are expressed in IBD patients, and a relation between polymorphisms of lncRNAs and IBD has been found in Genome Wide Association Studies (GWAS) [51]. The majority of IBD loci are in non-coding intergenic and intronic regions, and most of them overlap with regulatory zones, suggesting they may exert an influence on gene regulation [52].

The transcriptomic profile of colonic biopsies was analysed using gene expression microarrays [53]. The anti-sense lncRNAs DPP10-AS1, ANRIL and DIO3OS were downregulated in inflamed tissue from both CD and UC patients. Among them, ANRIL showed a 2.97-fold downregulation in inflamed CD compared to the control group. This study also showed that lncRNA expression profiling could be used to stratify both active and non-active CD and UC [53]. Furthermore, several years later it was demonstrated that ANRIL downregulation was associated with higher CD activity and positively correlated with anti-inflammatory cytokine levels [54]. Taken altogether, these studies indicate that lncRNA signatures may be used as predictive IBD biomarkers.

The expression of the lncRNAs MALAT1 and ANRIL was studied in colonic mucosa tissues from patients with UC and controls [55]. In this study, the expression of both lncRNAs was significantly and positively correlated in UC patients. Moreover, MALAT1 was upregulated in UC and promoted apoptosis of colonic endothelial cells by upregulating ANRIL [55]. These results strongly suggest an association between MALAT1 and ANRIL in UC. The role of MALAT1 in the regulation of proinflammatory cytokines such as IL-6 in IBD was also analysed [56]. In this study, IL-6 expression was shown to be higher in biopsy samples from IBD patients than in normal controls, and MALAT1 expression was also increased in IBD patients, without differences between CD and UC. These results suggest that MALAT1 plays a role in IL6 upregulation in IBD patients and point to MALAT 1 as a potential biomarker for IBD. MALAT 1 overexpression may dysregulate the production of TNF-alpha and IL6, which are involved in IBD [56].

Some studies focused only on one lncRNA. In this regard, Qiao et al. investigated the lncRNA DQ786243 by RT-qPCR, finding that this lncRNA could be related to severe CD and affected the expression of the CREB and Foxp3 genes [57]. Elamir et al. [58] studied the lncRNA THRIL in IBD; this lncRNA is related to innate immunity through the regulation of TNF-α. THRIL was upregulated in patients with IBD, compared to controls. This dysregulation involves the NFκB pathway, which increases the levels of proinflammatory cytokines linked to non-specific inflammatory responses in the intestine [58].

Sosnovski et al. [59] investigated the lncRNA GATA6-AS1, which is specifically expressed in the gut epithelium. The reduction in the expression of this molecule was associated with the worsening of UC. In addition, GATA6-AS1 reduction was associated with altered mitochondrial metabolic function. These results suggest that epithelial specific regulation of mitochondrial function by this lncRNA may be involved in the pathogenesis of IBD [59].

LncRNAs could work as diagnostic biomarkers for IBD. The lncRNAs DIO3OS, LINC01272 and KIF9-AS1 were selected by a certain study because of their differential expression in IBD [60], and their expression was assessed in both in tissue and plasma samples from patients with CD and UC and controls. Expression of LINC01272 and KIF9-AS1 was higher in CD and UC tissue samples compared to controls, whereas the expression of DIO3OS was lower in IBD samples compared to controls. Results in plasma were similar: LINC01272 and KIF9-AS1 were upregulated, while DIO3OS was downregulated in patients with IBD [60]. Furthermore, the expression of these lncRNAs in IBD tissue and plasma showed a positive correlation. The area under the receiver operating characteristic (ROC) curve (AUC) was higher than 0.75 for the upregulated lncRNAs in the comparisons of CD vs. controls, and UC vs. controls. In the case of DIO3OS, AUC was higher than 0.75 only for CD vs. controls. Therefore, expression levels of these three lncRNAs in tissue and plasma samples of IBD patients may have a potential value for IBD diagnosis [60].

The lncRNA GAS5 is involved in the regulation of the tissue injury mediators matrix metalloproteinases (MMPs) [61]. The role of GAS5 in regulating the expression of MMP2 and MMP9 was studied in IBD. Expression of this lncRNA was lower in inflamed areas of biopsies from drug-naive patients compared to adjacent non-inflamed tissue, while MMP2 and MMP9 levels were increased in inflamed biopsies [61]. These results confirmed the previously reported association of metalloproteinases with IBD in children [62]. In addition, expression of the lncRNA GAS5-AS1 (GAS5 antisense) was also downregulated in inflamed biopsies compared to non-inflamed tissue in children with IBD [63]. Therefore, these lncRNAs might represent possible biomarkers for IBD.

Another important lncRNA for IBD is ROCKI. There is a genetic link between ROCKI activity in blood monocytes and the risk of inflammation. Thus, some genetic variants affecting ROCKI expression are linked to a reduced risk of IBD [47].

The lncRNA CRNDE is upregulated in UC patients, compared to controls. This lncRNA plays a role in regulating pathways such as proliferation, apoptosis and metastasis but also plays a role in inflammatory pathways. In UC, CRNDE upregulation may increase inflammation by promoting the NF-kB pathway [64].

A remarkable link has been described between intestinal inflammation and cancer initiation. The lncRNA CCAT1 is increased in colon adenocarcinoma and promotes cancer cell proliferation. In addition, its expression is increased in inflamed colonic tissues and this promotes IBD by destroying the intestinal barrier function. Further studies are required to determine the potential of this lncRNA as a therapeutic target for IBD [65].

### 3.3. Untargeted Studies in Human IBD

In recent years, RNAseq techniques have acquired relevance in the field of transcriptomics, providing a tool to characterize the complete RNA landscapes of a great variety of biological samples.

The expression of lncRNAs and mRNAs was characterized by RNAseq in samples of normal intestinal tissue and CD mucosa and the top ten up- and downregulated lncRNAs were clustered [66]. Later, the differential expression of these lncRNAs between CD and control samples was verified by RT-qPCR. Results showed a differential expression in CD mucosa of lncRNAs potentially involved in the immune response [66].

Haberman et al. [67] conducted a study including 139 CD patients with clinically affected ileum and 38 non-IBD controls. In the discovery phase, they found 3022 differentially expressed genes (FDR < 0.05). They carried out an unsupervised hierarchical clustering that identified groups of patients with similar ileal gene expression profiles; one of these groups was the control group and the other group included most of the CD patients. In a principal components analysis, the three top dimensions showed that most control patients were separated from most of the CD patients [65]. In addition, in a supervised machine learning analysis with Support Vector Machine (SVM) which classified patients based on differential biological data, the accuracy was more than 80% in the discovery and independent validation groups. The correlation between lncRNA expression and tissue injury suggests that these lncRNAs could be used as targets for future interventions [67].

Microarrays are also useful in performing untargeted studies, although only in cases with a limited number of lncRNAs. Accordingly, Chen et al. used microarrays and RT-qPCR verification to determine the profile of plasma lncRNAs in CD patients. This study showed that microarrays combined with further bioinformatics analysis could help to correctly categorize subjects into CD and control groups according to their lncRNA profile, thus supporting the idea of using lncRNAs as a non-invasive method for CD diagnosis [66].

Recently, Braga-Neto et al. [68] studied ileal biopsies from 22 CD patients and 13 controls. They sequenced RNA from lamina propria CD4+T cells and detected 6402 long intergenic non-coding RNAs (lincRNAs). Analysis of lincRNAs differentially expressed in CD identified a new lincRNA called XLOC_000261, one potentially involved in IBD pathogenesis, that negatively regulates the transcription factor RORγt in Th17 cells. These results suggest that the novel lincRNA XLOC_000261 may play a role in regulating the immune system in CD [68].

### 3.4. LncRNAs and Response to Treatment in IBD Patients

Corticosteroids are widely used in IBD treatment. However, there is great variability in the response to glucocorticoids, as some patients are resistant to treatment while others respond quickly. Therefore, markers of glucocorticoid resistance are needed to improve treatment. The expression level of the lncRNA GAS5 in peripheral blood mononuclear cells (PBMCs) at the time of diagnosis and after 4 weeks of steroid treatment was able to discriminate between good and poor response to glucocorticoids in IBD patients [69]. This result suggests that GAS5 could be a potential marker of glucocorticoid resistance.

The lncRNA ANRIL has been associated with infliximab treatment response in CD [54]. Patients with active CD under infliximab treatment showed an increased expression of ANRIL compared to baseline (patients before treatment). This increased expression associated with treatment response could be explained by the anti-inflammatory role of ANRIL [54].

### 3.5. LncRNAs as Therapeutic Targets in Human IBD

Deregulated miRNAs and lncRNAs are involved in pathways that regulate immune responses (NF-kB signalling pathway) and influence the activity of Th17 cells. Therefore, lncRNAs, as well as certain miRNA/lncRNA pairs, may be considered as therapeutic targets for IBD [70].

Some studies have shown a connection between the intestinal epithelial barrier and IBD. Injury to the gut epithelial barrier is associated with inflammation and the development of UC. In this regard, expression of the lncRNA H19 seems to correlate with epithelial barrier function and development of UC [71]. Increased H19 expression correlates with decreased vitamin D receptor expression in UC tissues. Since H19 overexpression has a deleterious effect on intestinal barrier function, H19 and vitamin D receptor may be considered as therapeutic targets for UC [71]. In addition, H19 may discriminate between UC patients with moderate activity and those in remission [72], and it shows a high diagnostic accuracy in IBD [73]. This lncRNA can be detected in extracellular vesicles (EVs), which are small lncRNA-enriched vesicles that play an important role in intercellular communication. H19 was detected in plasma EVs and allowed distinguishment between patients with active IBD and those in remission. Taken altogether, the aforementioned results confirm that H19 is a potential good IBD biomarker that could be used to diagnose active IBD [74].

The UC-associated lncRNA BC012900 modulates intestinal epithelial cell susceptibility to apoptosis. Wu et al. collected colon biopsies from patients with different gastrointestinal alterations and healthy controls and used microarrays to assess the expression of lncRNAs and mRNAs [75]. Overall, 455 lncRNAs differentially expressed between active UC patients and controls were identified, and the most increased and decreased of those were validated with RT-qPCR. Among the selected lncRNAs, BC029135 was unique in active UC, showing responses to stimuli that can compromise the intestinal barrier function [75].

Lastly, CNN3-206 expression is increased in intestinal lesions of CD patients, leading to increased Caspase 10 through reduction of miR212 levels. Activation of this CNN3-206-miR212-Caspase 10 regulatory network increases apoptosis, migration and invasion in intestinal epithelial cells [76]. These results suggest that CNN3-206 could be a potential therapeutic target for CD treatment.

The results and main conclusions of the most recent human studies on the role of lncRNAs in IBD are summarised below for CD (Table 1) [54,57,66,67,68,77], UC (Table 2) [55,71] and both CD and UC (Table 3) [53,56,58,59,60,61,63,64,69,72,74,75].

## 4. Conclusions

The study of ncRNAs may provide relevant information about the pathogenesis of several diseases, such as IBD. LncRNAs show highly tissue-specific expression patterns and can be isolated from total RNA present in many biological samples, including blood. The discovery of the mechanisms of action and the roles played by these molecules in many diseases—including IBD—is becoming increasingly relevant. Despite the efforts of investigators to elucidate a cluster of lncRNAs that could be used to differentiate among CD and UC, at present, there is not a single lncRNA that could be considered a relevant biomarker of disease.

Further studies are necessary to unravel the real function of lncRNAs in IBD. The study of lncRNAs may be more relevant for obtaining a better understanding of IBD pathophysiology than for unraveling novel biomarkers. Furthermore, lncRNAs could work as potential therapeutic targets for IBD. Therefore, understanding the role of lncRNAs in IBD is potentially relevant for the management of this disease and warrants further studies.

## Figures and Tables

**Table 1:** Relevant studies on human lncRNAs and Crohn’s disease.

Author	Publication Year	lncRNA	Sample Size of the Study	Biological Samples and Methods	Conclusions
Qiao et al. [57]	2013	DQ786243	Active CD = 11 Quiescent CD = 8 HC = 9	PBMCs. RT-qPCR.	DQ786243 could be related to severe CD.
Chen et al. [77]	2016	1988 dysregulated lncRNAs,10 were selected according to results from Mirza et al. [53]	CD = 12 HC = 12	Plasma. Microarrays, RT-qPCR.	LncRNAs as non-invasive method for CD diagnosis.
Haberman et al. [67]	2018	459 differentially expressed lncRNAs	Active CD = 139 HC = 39	Ileum biopsies. RNAseq., RNA in situ hybridization RT-qPCR.	LncRNAs are potential targets for CD activity.
Ge et al. [54]	2019	ANRIL	Active CD = 42 Quiescent CD = 59 HC = 67	Intestinal mucosa. qPCR.	ANRIL is associated with CD and disease activity. ANRIL increases after infliximab treatment.
Li and Shi [66]	2020	The top 8 down- and upregulated lncRNAs	Active CD = 60 HC = 60	Ileum biopsies. Microarrays, RT-qPCR.	lncRNAs could be targets for CD activity.
Braga-Neto et al. [68]	2020	XLOC_000261	Active CD = 22 HC = 13	Terminal ileal biopsies. RNAseq.	Novel lincRNA for CD studies.

CD: Crohn’ s disease; HC: healthy controls; PBMCs: peripheral blood mononuclear cells; lncRNA: long non-coding RNA; RT-qPCR: real time quantitative polymerase chain reaction; lincRNA: long intergenic non-coding RNA.

**Table 2:** Relevant studies on human lncRNAs and ulcerative colitis.

Author	Publication Year	lncRNA	Sample Size of the Study	Biological Samples and Methods	Conclusions
Chen et al. [77]	2016	H19	UC = 12	UC inflamed tissues and paired normal tissues. RT-qPCR.	H19 overexpression exerts a destructive effect on the intestinal barrier. H19 decreases expression of vitamin D receptor in UC.
Zhu et al. [55]	2020	MALAT1, ANRIL	UC = 76 HC = 76	Plasma and tissue biopsies. RT-qPCR.	MALAT1 is upregulated in UC and promotes apoptosis of colonic endothelial cells by upregulating ANRIL.

UC: ulcerative colitis; HC: healthy controls; RT-qPCR: real time-quantitative polymerase chain reaction.

**Table 3:** Relevant studies on human lncRNAs and both Crohn´s disease and ulcerative colitis.

Author	Publication Year	lncRNA	Sample Size of the Study	Biological Samples and Methods	Conclusions
Mirza et al. [53]	2015	DPP10-AS1, ANRIL, DIO3OS	CD = 13 UC = 20 HC = 12	Tissue biopsies. Microarrays.	LncRNA expression profiling allows stratification of CD and UC patients.
Wu et al. [75]	2016	BC012900	Active UC = 16 Quiescent UC = 15 Active CD = 5 IBS = 5 HC = 5	Colon biopsies. Microarrays, RT-qPCR.	BC012900 is highly expressed in active UC and modulates intestinal epithelial cell susceptibility to apoptosis.
Wang et al. [60]	2018	DIO3OS, LINC01272, KIF9-AS1	CD = 84 UC = 84 HC = 84	Tissue biopsies and plasma. RT-qPCR.	LINC01272 and KIF9-AS1 are upregulated and DIO3OS is downregulated in IBD patients.
Lucafò et al. [69]	2018	GAS5	CD = 16 UC = 3	PBMCs. RT-qPCR.	GAS5 could be a novel pharmacogenomic marker for glucocorticoid therapy.
Lucafò et al. [61]	2019	GAS5	CD = 13 UC = 12	Paired inflamed and non-inflamed tissue biopsies in each patient. RT-qPCR.	GAS5 expression is lower in areas of inflamed tissue compared to adjacent non-inflamed areas.
Elamir et al. [58]	2022	THRIL	CD = 70 UC = 70 HC = 70	Serum. qRT-PCR.	THRIL is implicated in inflammatory responses, and it is upregulated in IBD.
Sosnovski et al. [59]	2023	GATA6-AS1	673 samples from PROTECT, SEEM and SOURCE studies	Tissue. qRT-PCR.	GATA6-AS1 reduction is associated with the worsening of IBD.
Bajestan et al. [56]	2023	MALAT1	CD = 13 UC = 20 HC = 20	Intestinal tissue. qRT-PCR.	MALAT1 gene could be a target in IBD diagnosis and treatment. MALAT1 may be associated with proinflammatory cytokines.
Curci et al. [63]	2023	GAS5-AS1	26 IBD	Colonic biopsies. qRT-PCR.	Intestinal inflammation promotes GAS5-AS1 downregulation. This lncRNA is important for the immune system.
Shaker et al. 2023 [72]	2023	H19	CD = 32 UC = 35 HC = 30	Serum. qRT-PCR.	H19 is overexpressed in IBD and is a good candidate for IBD diagnosis and treatment.
Kazemifard et al. [64]	2023	CRNDE	42 IBD 47 HC	Tissue and blood sample collection. RNAseq.	CRNDE is associated with inflammation pathways, and it could be a marker of inflammation in IBD.
Heydari et al. [74]	2024	H19	22 active IBD 14 remission IBD 10 RA 14 IBS	Tissue and plasma. qRT-PCR.	H19 distinguishes among all groups of patients, and it is a good candidate for diagnosis of active IBD.

CD: Cronh’s disease; UC: ulcerative colitis; IBD: inflammatory bowel disease; IBS: irritable bowel syndrome; PBMCs: peripheral blood mononuclear cells; RT-qPCR: real time-quantitative polymerase chain reaction; RA: rheumatoid arthritis; RNAseq: RNA sequencing.

## Data Availability

No new data have been generated.
